# A review of proton beam therapy’s role in glioma management

**DOI:** 10.1097/MD.0000000000043071

**Published:** 2025-07-04

**Authors:** Nicholas Aderinto, Gbolahan Olatunji, Emmanuel Kokori, Doyin Olatunji, Oluwatobi Omoworare, Adetola Emmanuel Babalola, Chimezirim Ezeano, Damilare Adewale Olusanya, David Timilehin Isarinade, Aminat Akinoso, Adeola Akinboade, Abdulrahmon Moradeyo, Naana Nkrumah-Ababio

**Affiliations:** a Department of Medicine, Ladoke Akintola University of Technology, Ogbomoso, Nigeria; b Department of Medicine and Surgery, University of Ilorin, Ilorin, Nigeria; c Department of Health Sciences, Western Illinois University, Macomb; d Department of Medicine and Surgery, Lagos State University, Lagos, Nigeria; e Faculty of Dentistry, College of Medicine, University of Ibadan, Ibadan, Nigeria; f University of North Texas, Health Science Centre, Fort Worth, Texas; g West Suffolk Hospital, Bury St. Edmunds, United Kingdom; h Alberta Health Services, Calgary, Alberta, Canada.

**Keywords:** brain tumors, glioma, neurocognitive function, proton beam therapy, quality of life, radiotherapy

## Abstract

Gliomas pose significant therapeutic challenges due to limited survival and risks of treatment-related toxicity. Proton beam therapy (PBT) offers a precise radiation delivery method, minimizing damage to healthy brain tissue compared to conventional radiotherapy. This review synthesizes findings from 15 studies (2000–January 2024) from PubMed, Google Scholar, Science Direct, Cochrane Library, and Directory of Open Access Journals. PBT significantly reduces neurocognitive decline and enhances quality of life while achieving comparable or superior survival outcomes across various glioma types, including low-grade gliomas and glioblastomas. Notable benefits include improved verbal memory, stable intellectual functioning, and reduced high-grade toxicity. However, challenges such as neuroendocrine deficiencies and increased radiation necrosis highlight the need for optimized protocols. Future research should focus on comprehensive outcome assessments, proactive management of adverse effects, and improving PBT accessibility through cost reduction and technological advancements. PBT holds transformative potential in glioma management, balancing effective tumor control with preservation of neurological function, positioning it as a valuable neuro-oncology treatment option.

## 1. Introduction

Gliomas are the most common primary brain tumors.^[1]^ Out of 250,000 new cases of primary brain malignancies diagnosed each year, gliomas account for 77%.^[2]^ Gliomas are graded by the World Health Organization (WHO) from 1 to 4 based on their aggressive potential in the near term. Grades 3 and 4 are considered high grades with poor prognoses.^[3]^ High-grade gliomas represent a range of heterogeneous tumors with diverse biological profiles, displaying strong capabilities for early treatment resistance development.^[4]^ With a <5% 5-year survival rate for its most common histologic type, glioblastoma, gliomas cause significant mortality and morbidity.^[5]^

Due to numerous factors, including the site of the tumor and poorly demarcated tumor borders, complete resection of the tumor is often unattainable. Consequently, radiotherapy has become a mainstay in treating gliomas.^[6]^ This is often carried out postoperatively or as the primary treatment when surgery cannot occur. Exposure of normal brain tissue to radiation negatively affects the brain’s plasticity and functions.^[3]^ Radiation injury can be early, acute, subacute, and late, with the latter being progressive and irreversible, leading to neurocognitive dysfunction.^[7]^ Tumor relapse and progression are also common, especially amongst high-grade gliomas, even after radiation therapy.^[8]^

Proton beam therapy (PBT) is a form of radiotherapy that uses protons rather than photons, used in conventional radiotherapy (CRT).^[9]^ Proton beams, known for their sharper dose gradient compared to conventional photons, allow precise radiation delivery with potentially reduced toxicity to surrounding healthy tissues.^[6]^ This dose escalation is beneficial in some instances, which is not feasible in CRT.^[10]^ PBT’s ability to spare normal brain tissue from unnecessary radiation holds tremendous potential in reducing the cognitive and neurological side effects often associated with traditional treatments. Moreover, the potential to minimize radiation exposure to healthy tissues extends beyond immediate concerns. PBT may reduce long-term side effects, thus enhancing the overall quality of life for glioma patients posttreatment.

The growing body of evidence from preliminary studies and ongoing clinical trials increasingly substantiates the efficacy and safety of PBT in treating gliomas.^[9,11]^ Early findings suggest that PBT offers comparable tumor control rates to conventional photon therapy, while also significantly reducing treatment-related adverse effects, such as cognitive impairment and neurological deficits. This review aims to evaluate the current role of PBT in treating gliomas, integrate existing research findings, and offer a comprehensive analysis of its clinical outcomes. The review addresses the challenges in optimizing proton therapy protocols, emphasizing the need for individualized treatment strategies based on tumor type, location, and patient-specific characteristics. Furthermore, the review highlights the need for further investigation into the long-term effects of proton therapy, including survival outcomes, recurrence patterns, and the impact on overall quality of life for glioma patients.

## 2. Methodology

**Figure 1.**
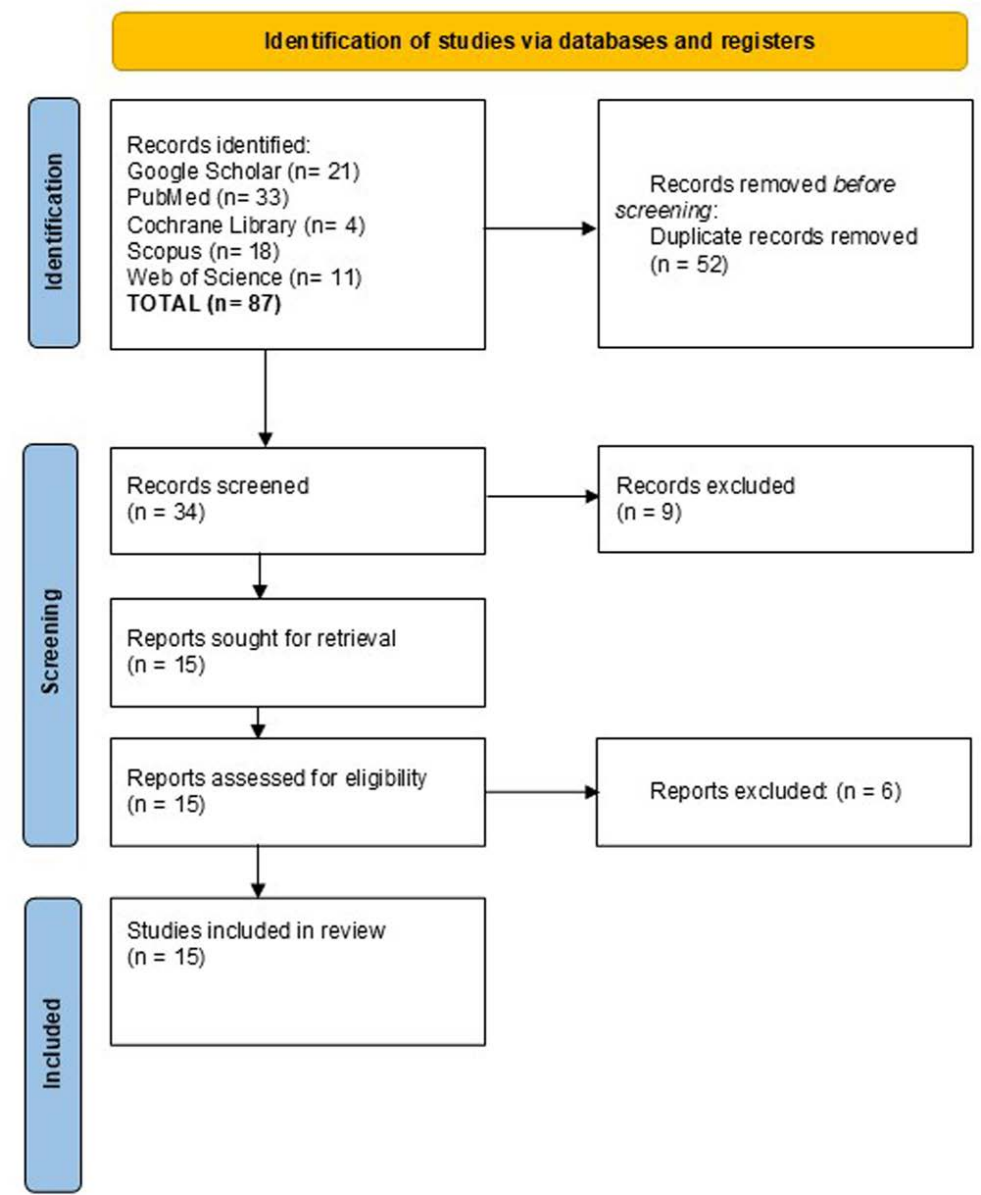
PRISMA flow diagram.

This narrative review retrieved relevant literature from several prominent academic databases, including PubMed, Google Scholar, SCOPUS, Cochrane Library, and the Web of Science. The search strategy was designed to maximize the retrieval of pertinent studies by combining controlled vocabulary (MeSH terms) and free-text keywords. The primary search terms included “Proton therapy” and “Glioma,” with variations in phrasing (e.g., “proton beam therapy,” “proton radiation”) to ensure broader capture of relevant articles. These terms were combined using Boolean operators such as AND, OR, and NOT to refine search results and focus on studies that directly addressed the research questions (Fig. 1).

Special settings were applied during the search process for each database to ensure consistency and thoroughness across platforms. In PubMed, for example, filters were applied to include only human studies published in English and available in full-text. Google Scholar and Science Direct were searched with a focus on peer-reviewed articles, and Cochrane Library was filtered for systematic reviews and clinical trials. The search period was set from 2000 through January 2024, capturing recent and relevant data.

Inclusion criteria required studies to focus on proton therapy as a glioma therapeutic modality, specifically comparing PBT to conventional photon radiation therapy. Only original research articles were considered; we excluded non-primary research such as letters, opinion pieces, editorials, preprints, comments, and reviews. Studies not published in English or lacking full-text access were also excluded.

Data synthesis and extraction were done using an Excel spreadsheet (version 2401). Two independent reviewers (AB and MA) performed the initial screening of articles, applying the inclusion and exclusion criteria. Any disagreements or uncertainties were resolved through discussion with a third independent reviewer (NA) to ensure the integrity and reliability of the selection process.

## 3. Glioma overview

Gliomas are primary brain tumors that arise from the glial stem or progenitor cells of the central nervous system.^[12]^ These tumors can develop in various regions of the brain and spinal cord, with the brain being the most common site of occurrence.^[12]^ Gliomas affect individuals across all age groups, though they are more prevalent in adults, with a higher incidence observed in males than females.^[13]^

Gliomas can be broadly categorized based on their malignancy and pathological characteristics. The classification begins with the distinction between circumscribed gliomas and diffuse gliomas.

- Circumscribed gliomas are benign, typically well-defined, and can often be effectively treated with total surgical resection.^[13]^ They are generally less aggressive and have a better prognosis.
- Diffuse gliomas, on the other hand, are malignant and exhibit aggressive growth patterns that prevent complete surgical resection from providing a cure. These gliomas infiltrate surrounding brain tissue and constitute about 75% of primary malignant brain tumors.^[14]^

Among the diffuse gliomas, the level of malignancy further differentiates several subtypes:

1. Glioblastoma is the most malignant and aggressive form of glioma, often associated with poor prognosis and rapid progression.
2. Astrocytomas, including low-grade variants like pilocytic astrocytoma, are typically less aggressive. Pilocytic astrocytomas are considered the least malignant within this group.^[15]^

Gliomas can also be classified based on their molecular and genetic markers, which have become increasingly important in guiding treatment decisions. The fifth edition of the World Health Organization’s Classification of Tumors of the Central Nervous System (WHO CNS5) classifies gliomas into 4 broad categories^[16]^:

1. Adult-type diffuse gliomas: these include glioblastomas and diffuse astrocytomas that typically occur in adults.
2. Pediatric-type diffuse low-grade gliomas: these tend to be less aggressive and are often found in children.
3. Pediatric-type diffuse high-grade gliomas: these are more aggressive and carry a poorer prognosis, similar to their adult counterparts.
4. Circumscribed astrocytic gliomas: these include pilocytic astrocytomas and other less aggressive forms, often confined to a single region.

Symptoms of gliomas vary depending on their location and grade, but commonly include headaches, seizures, transient loss of consciousness, tingling sensations, and weakness. In severe cases, patients may progress to a comatose state.^[15]^

Radiation therapy has been a cornerstone in the management of gliomas for several decades. Imaging techniques such as computed tomography and magnetic resonance imaging are essential in the standard radiotherapy planning for high-grade gliomas.^[4]^ Early initiation of radiation therapy has been shown to improve seizure control, although it does not significantly enhance overall survival (OS).^[3,17]^

Conventional photon-based external beam radiation therapy is the most commonly used form of radiotherapy. It uses high-energy photons to target and destroy cancerous cells. However, a significant challenge with photon therapy is its nonselective nature, which irradiates surrounding healthy tissues, leading to potential side effects.^[14]^

Brachytherapy, a form of internal radiation therapy where radioactive sources are placed directly within or near the tumor, is another treatment option. While it allows for more localized radiation delivery, its application in glioma treatment is often limited by factors such as tumor location, size, and the invasive nature of the procedure.^[5,18]^ Although both photon therapy and brachytherapy are effective in controlling glioma progression, there is an ongoing need for treatment strategies that achieve better tumor control while minimizing the impact on normal brain function.

## 4. PBT

PBT is a growing alternative to standard photon radiotherapy as a treatment modality for several cancer types.^[19]^ PBT uses proton beams to irradiate cancer cells due to the unique physical features of proton particles.^[20]^ Protons possess heavier subatomic particles that deliver their energy more precisely with little dispersion to surrounding normal tissues.^[21]^ This physical characteristic offers PBT a dosimetric advantage, reducing long- and short-term adverse effects.^[22]^ In addition to its physical characteristics, PBT is described in terms of its relative biological effectiveness (RBE).^[23]^ The RBE generally describes the effectiveness of radiation modalities in inducing biological effects in target cells. An RBE of 1.1 is clinically used for protons, and doses are typically corrected for RBE, reported in units of cobalt gray equivalents or gray equivalents (GyE) to ensure comparability with photon (x-ray) dose prescriptions.^[19,20]^ RBE is also relevant in determining the effective dose for Proton Beam Therapy.^[19]^

The physical characteristics of protons, their large size and acceleration, allow for the delivery of a large dose within the final millimeters of their range, described as the Bragg Peak.^[21]^ The characteristics of PBT described allow for dose conformity, enabling the delivery of more precise doses to the areas of interest and sparing normal tissue.^[23]^ Subsequent deposition of the proton energy causes ionization of molecules, leading to radiation damage in the DNA of the target cell.^[22]^

The benefits of PBT are particularly evident when considering the different types of gliomas, as the therapy’s precision enables tailored treatment approaches.^[22]^ For low-grade gliomas (LGGs), which typically affect younger individuals with longer life expectancy, PBT offers substantial advantages by reducing neurocognitive side effects and sparing healthy tissue, which is critical for preserving long-term quality of life.^[22]^ In contrast, high-grade gliomas, such as glioblastomas, are aggressive and often located near essential structures of the brain, like the brainstem and optic nerves. These tumors require highly conformal radiotherapy to maximize tumor control while minimizing damage to adjacent healthy tissues, a requirement well met by PBT.^[18]^ PBT provides several physical and clinical advantages over conventional photon radiotherapy. One of its key features is the low entrance and exit dose, allowing energy to be deposited at a specific depth due to the Bragg Peak effect.^[22]^ This results in a significant dosimetric advantage, reducing the integral dose to surrounding vital neurological structures while maintaining sufficient coverage of the target volume.^[24,25]^ These properties contribute to reducing adverse neurocognitive and neuroendocrine effects commonly associated with traditional radiotherapy,^[10,25]^ even in patients with preexisting cognitive deficits.^[10]^ Furthermore, PBT is generally well-tolerated, with no significant decline in overall quality of life reported. This is particularly beneficial for patients undergoing re-irradiation for recurrent tumors and for younger patients with LGGs who are expected to live longer.^[10]^

Despite these benefits, the widespread use of PBT is limited by its high cost and the substantial financial resources required to establish and maintain treatment facilities. These barriers pose significant challenges for patients seeking access and restrict research opportunities, particularly in evaluating long-term toxicities associated with proton therapy.^[10]^

## 5. Analysis of studies on PBT

Thirteen studies met the inclusion criteria for this review, as shown in Table 1. The studies included silico trials (3), prospective trials (4), randomized controlled trials (4), and retrospective studies (4). They focused on different gliomas, including low-grade gliomas, primary glioblastoma, and recurrent glioblastoma. The range for radiation doses administered in the included studies was 50 to 75 Gy (Gray). Considering all the studies, the mean radiation dose was approximately 60 Gy.

**Table 1:** Summary of included studies evaluating proton beam therapy in the management of gliomas.

Authors and reference	Study design	Sample size	Glioma type	Intervention	Comparison	Follow-up duration	Results: tumor control	Results: neurocognitive outcomes	Adverse events
Jhaveri et al^[38]^	Retrospective NCDB analysis	49,575	WHO Grade I–IV	PBT (n = 170): dose/fractionation varied; OS evaluated via Cox models and propensity score weighting	Photon therapy (XRT, n = 49,405)	Median 62.1 mo	PBT associated with longer median OS (45.9 vs 29.7 mo, *P* = .009); 5-year OS (46.1% vs 35.5%, *P* = .016)	Not assessed	Not reported
Mohan et al^[30]^	Randomized Phase II Trial	84	GBM	PTV-50: 50 Gy (RBE); PTV-60: 60 Gy (RBE) in 30 fractions	Same plan for photons and protons (RBE 1.0 vs 1.1)	1 mo	Not primary endpoint; PBT reduced severe lymphopenia	Reduced irradiated brain volumes; potential for less lymphopenia	Not applicable
Eekers et al^[26]^	Silico ROCOCO trial	25	Grade 2 LGG	Dose: 50.4 Gy in 28 fractions	RBE 1.0 (photons) vs 1.1 (protons)	Not applicable	Not applicable	Posterior cerebellar radiation negatively affects long-term neurocognition	Not applicable
Scartoni et al^[32]^	Cross-sectional study	33	Not specified	36 GyRBE in 18 fractions; 25% with concomitant TMZ	Prior photon RT: 60 Gy in 30 fractions + TMZ	3 mo	Not reported	Not reported	Not reported
Willmann et al^[37]^	Retrospective & prospective longitudinal	89	Grade 1 or 2 LGG	Total dose: 54 Gy	Not applicable	6 yr	Long-term tumor control and survival in LGG	General stability in cognitive function	82% grade 1–2 toxicity; 1 grade 3, 2 grade 4 toxicities
Hauswald et al^[36]^	Retrospective study	19	Grade I & II glioma	Median dose: 54 GyE (range 48.6–54 GyE) in 1.8 GyE fractions	Not specified	5 mo	Not reported	Not reported	13 focal alopecia, 6 mild fatigue, 4 no acute effects
Tabrizi et al^[33]^	Prospective study	20	Grade II	54 Gy (RBE) in 30 fractions	Not applicable	5 yr	Not reported	Majority retained stable cognitive and neuroendocrine function	Fatigue, headache, alopecia
Matsuda et al^[29]^	Retrospective study	235	Grade I–III	PBT: 96.6 Gy (RBE) or CRT: 60 Gy in 56 fractions	Photon: 60 Gy in 30 fractions	PBT: 28.3 mo; CRT: 21.2 mo	Not reported	Not reported	Alopecia, dermatitis, otitis, radiation necrosis
Sherman et al^[27]^	Observational study	20	Grade II	54 Gy (RBE) at 1.8 Gy (RBE)/fraction over 6 weeks	Not applicable	5.1 yr	Tumor location affects function (left worse on verbal scores)	Conformal advantage of PRT may preserve cognition	Not reported
Shih et al^[10]^	Prospective trial	20	Grade II	54 Gy (RBE) at 1.8 Gy (RBE)/fraction over 6 weeks	20 Gy comparator	5.1 yr	Not reported	No evidence of cognitive or QOL decline	Fatigue, alopecia, headache, scalp erythema
Petruccelli et al^[28]^	Cohort study	23	Low-grade glioma	Mean dose reduction to brain: Left temporal lobe (1490–1092 cGy), brain (750–451 cGy) in EQD2	Not applicable	Not applicable	RT effective but with neurocognitive decline risk	Lower brain dose with PBT linked to lower cognitive impairment risk	Not reported
Bronk et al^[31]^	Retrospective study	99	Grade II or III	PBT with passive scatter or scanning beam; RBE 1.1	Not applicable	46 mo	Not reported	Not reported	Not reported
Maquilan et al^[35]^	Cohort study	23	Grade I–III	54 Gy (RBE) at 1.8 Gy (RBE)/fraction; weekly and serial follow-up	Not applicable	1 mo	Not reported	Not reported	Anorexia, fatigue, nausea, headache, insomnia
Mizumoto et al^[34]^	Case report	81	GBM	46.2 GyE in 28 fractions to contrast-enhanced areas 6 h post x-ray RT	50.4 Gy to T2-high areas	70.9 mo	High-dose PBT may control GBM if infiltration fully covered	Not reported	Nausea, headache, alopecia
Brown et al^[39]^	Phase II RCT	90 enrolled (67 evaluable)	Newly diagnosed glioblastoma	Proton radiotherapy (PT)	Intensity-modulated radiotherapy (IMRT)	Time to cognitive failure (primary), OS, PFS, toxicity, PROs	No significant difference in cognitive failure (HR 0.88; *P* = .74), PFS (HR 0.74), or OS (HR 0.86); PT associated with significantly reduced fatigue (24% vs 58%; *P* = .05) and fewer grade ≥ 2 toxicities (mean 0.35 vs 1.15; *P* = .02); reduced radiation dose to normal structures		

CRT = conventional radiotherapy, GBM = glioblastoma, LGG = low-grade glioma.

### 5.1. Positive outcomes

Eekers et al^[26]^ reveal that proton therapy significantly reduces the mean dose to organs at risk compared to volumetric modulated arc therapy in LGG patients. The study suggests the potential for reduced neurocognitive decline and improved quality of life with PBT. Similarly, Sherman et al^[27]^ evaluated the neurocognitive effects of PBT in adults with low-grade glioma over 5 years. Patients exhibited stability in cognitive functioning over time. Tumour location influenced baseline performance, and there was greater improvement in verbal memory over time in patients with left hemisphere tumors. Shih et al^[10]^ focused on the tolerability and outcomes of proton therapy for low-grade gliomas. All 20 patients tolerated treatment without difficulty, and the median follow-up after proton therapy was 5.1 years. Intellectual functioning was within the normal range for the group and remained stable over time, with no overall decline in cognitive functioning. However, some patients did develop endocrinopathies following treatment. It is important to note that the study did not include a photon therapy comparator group, limiting the ability to directly attribute these findings to the advantages or disadvantages of proton therapy relative to conventional modalities.

Petruccelli et al^[28]^ compared proton therapy to photon therapy in adults with brain tumors. Proton therapy showed lower estimated probabilities of impairment in neurocognitive test scores compared to photon therapy. There was a reduction in the probability of impairment in the COWAT test from 6.8% to 5.4%. Similar results were seen in the WAIS-IV Coding test, which ranged from 5% to 4.1%. In addition, Matsuda et al^[29]^ compared high-dose PBT with conventional fractionated radiation therapy for newly diagnosed glioblastoma. The PBT group’s median OS was 28.3 months, while the median OS of the CRT group was 21.2 months. Acute radiation-related toxicities were equivalent in both groups. Mohan et al^[30]^ demonstrated that proton therapy reduces the likelihood of high-grade radiation-induced lymphopenia in glioblastoma patients. Proton therapy reduced the likelihood of high-grade radiation-induced lymphopenia compared to x-ray therapy. Bronk et al^[31]^ compared the rate of pseudoprogression (PsP) after proton therapy to photon therapy for grade II and III gliomas. The study finds no significant difference in PsP rates between proton and photon therapy, with PsP occurring earlier in proton-treated oligodendroglioma patients.

Scartoni et al^[32]^ investigated proton therapy re-irradiation in large recurrent glioblastoma patients. The treatment was associated with improved stability in most preselected health-related quality of life domains. Global health improved over time with a maximum difference of 6 points between baseline and 3-month follow-up. Social functioning and motor dysfunction improved over time with a maximum difference of 8 and 2 points, respectively. Similarly, Tabrizi et al^[33]^ assessed proton radiotherapy’s long-term outcomes and late adverse effects for low-grade glioma patients. Proton therapy demonstrates favorable long-term morbidity outcomes, with stable cognitive and neuroendocrine function in the majority of patients. Most long-term toxicities develop within 2 years after radiation therapy. Additionally, Mizumoto et al^[34]^ evaluated the long-term survival after treatment of glioblastoma multiforme with hyperfractionated concomitant boost PBT. High-dose PBT shows control of glioblastoma pathogenesis, with long-term survivors exhibiting well-preserved remaining brain volume.

Maquilan et al^[35]^ contribute insights into the acute toxicity profile of proton therapy for low-grade gliomas and meningiomas. The study reports a favorable profile, with most patients experiencing mild fatigue, headache, insomnia, and a low rate of severe acute side effects. Also, Hauswald et al^[36]^ retrospectively assessed the feasibility and toxicity of proton therapy in patients with low-grade glioma. Proton therapy is completed as planned in all cases, with early side effects, such as mild alopecia, being predominant. In the Willmann et al^[37]^ study, patients treated with pencil-beam proton therapy achieved excellent long-term survival and tumor control, with exceptionally low rates of high-grade late toxicity and favorable quality of life and sexual health. Jhaveri et al^[38]^ analyzed NCDB data and found that glioma patients treated with PBT had significantly better median and 5-year survival than those receiving photon therapy, even after propensity score weighting. However, the small number of PBT patients (n = 170) and the study’s retrospective nature raise concerns about selection bias and unmeasured confounding, such as tumor molecular markers and performance status. These findings, while promising, require validation in prospective randomized trials.

### 5.2. Negative outcomes

Shih et al^[10]^ detected new endocrine dysfunctions primarily linked to direct hypothalamic-pituitary axis irradiation in 6 patients undergoing proton therapy. The progression-free survival rate declined from 85% at 3% to 40% at 5 years, showing the importance of monitoring for neuroendocrine sequelae following PT. Similarly, Matsuda et al^[28]^ reported that while PBT offered survival benefits, it was associated with an increased incidence of radiation necrosis compared to CRT.

Scartoni et al^[29]^ and Tabrizi et al^[33]^ independently reported neuroendocrine deficiencies in subsets of patients, particularly those receiving higher doses to the hypothalamic-pituitary axis. Most long-term toxicities in these cohorts emerged within 2 years of treatment. Mizumoto et al^[34]^ also noted radiation necrosis in 6 surviving patients, despite the absence of tumor recurrence, reinforcing concerns over late-onset adverse effects. Conversely, Maquilan et al^[35]^ reported a favorable acute toxicity profile for proton therapy in low-grade gliomas and meningiomas, with most side effects, such as mild fatigue, headache, and insomnia, resolving within 1 month posttreatment. Hauswald et al^[36]^ also found PT feasible in low-grade gliomas, with mild alopecia being the predominant side effect, usually associated with large treatment volumes and the anatomical location of target volumes.

Complementing these findings, a recent prospective phase II randomized trial by Brown et al^[39]^ compared proton therapy to intensity-modulated radiotherapy in patients with newly diagnosed glioblastoma. While proton therapy did not significantly delay cognitive failure compared to intensity-modulated radiotherapy, it demonstrated a more favorable toxicity profile. Notably, patients receiving PT experienced significantly fewer grade 2 or higher toxicities and lower rates of fatigue (24% vs 58%, *P* = .05). Despite no significant differences in progression-free survival, OS, or patient-reported outcomes at 6 months, the dosimetric advantages of PT led to lower radiation exposure to surrounding brain structures. These findings suggest that while PT may not yet provide clear cognitive or survival benefits in glioblastoma, it can reduce treatment-related morbidity, which is particularly important in optimizing long-term quality of life.

## 6. Challenges and future directions

While the studies presented shed light on both the positive and negative aspects of proton therapy for various brain tumors, several challenges and avenues for future exploration emerge. Understanding and addressing these challenges are crucial for refining the application of proton therapy and maximizing its benefits in neuro-oncology. One notable challenge is the need for more focus on clinical outcomes and long-term effects in the reviewed studies. Many investigations predominantly centered on dosimetric differences and specific adverse effects, leaving a gap in our understanding of the impact of proton therapy on patients’ quality of life, functional outcomes, and survival rates. Future research should prioritize comprehensive assessments to provide a holistic view of the treatment’s efficacy.

The neurocognitive and endocrine effects show the need to understand better how proton therapy influences cognitive functions and endocrine systems. Larger sample sizes and extended follow-up periods are essential to discern patterns, identify risk factors, and develop strategies to mitigate potential impairments. Additionally, proactive management approaches for detected dysfunctions should be a focus of future investigations. Studies such as Matsuda et al^[34]^ have highlighted an increased risk of radiation necrosis with high-dose PBT. Emerging literature highlights additional radiobiological complexities of PT, particularly related to radiation-induced contrast enhancement (RICE). Harrabi et al^[40]^ prospectively assessed 110 patients with low-grade gliomas treated with PRT and observed a substantially higher-than-expected incidence of asymptomatic RICE, especially at the distal edge of the proton beam. These lesions were strongly correlated with regions of increased linear energy transfer (LET), indicating that proton-induced brain injury may be underestimated when using a fixed RBE of 1.1. This concern is echoed in findings by Eulitz et al,^[41]^ who analyzed radiation-induced brain injuries in WHO grade 2 to 3 glioma patients treated with proton therapy. The study demonstrated a strong spatial correlation between radiation-induced brain injuries lesions, increased LET, and proximity to the periventricular region: a radiosensitive anatomical zone. Future directions should prioritize the development of effective treatment strategies for radiation necrosis, ensuring that this adverse effect does not compromise the therapeutic benefits of proton therapy. Advanced imaging techniques and targeted interventions play pivotal roles in this context.

Optimising proton therapy protocols remains an ongoing challenge. Tailoring treatment plans to individual patient characteristics, tumor types, and anatomical locations is essential for maximizing therapeutic efficacy while reducing the risk of toxicity. One promising avenue is LET-optimized proton therapy planning, which goes beyond conventional dose-based approaches by explicitly incorporating the biological impact of LET into treatment algorithms. Recent planning algorithm advancements enable biologically informed treatment plans that balance dose distribution with LET minimization in organs at risk.^[42]^ Further, McIntyre et al^[43]^ emphasized the importance of LET-guided intensity-modulated proton therapy and adaptive planning strategies, particularly for pediatric and central nervous system tumors where standard tissue tolerance is low. Their findings support the development of multi-objective optimization frameworks that consider physical dose and LET distributions, allowing clinicians to steer high-LET regions away from sensitive brain structures such as the brainstem and periventricular white matter. Therefore, integrating LET-based metrics into clinical treatment planning systems, advanced imaging, and real-time monitoring may mitigate the risks of RICE and late neurotoxicity. Future prospective studies are warranted to validate the clinical utility of LET-optimized planning and to refine RBE models for personalized proton therapy.

Cost considerations and limited accessibility impede the widespread adoption of proton therapy. Overcoming these challenges requires continued technological advancements to reduce treatment costs and the development of more compact proton therapy facilities. Collaboration between healthcare providers, researchers, and policymakers is essential to ensure broader access to this advanced treatment modality.

A critical step toward resolving the current uncertainties surrounding proton therapy is the conduct of well-designed, adequately powered randomized controlled trials. The recently closed NRG Oncology BN005 trial^[44]^ exemplifies this effort. BN005 directly compared the cognitive and clinical outcomes of proton versus photon radiotherapy in patients with IDH-mutant low- and intermediate-grade gliomas. The rationale behind this trial lies in the hypothesis that proton therapy may better preserve neurocognitive function by reducing radiation exposure to healthy brain tissue, particularly in eloquent regions, while acknowledging concerns about potential differences in tumor control due to variations in dose distribution and RBE. BN005 has completed accrual as of March 2024, and its outcomes are expected to provide high-quality evidence on whether the theoretical dosimetric advantages of proton therapy translate into meaningful clinical benefit. Until such data are available, decisions regarding the routine use of proton therapy in low-grade gliomas must be made cautiously, ideally within clinical trials or carefully selected patient cohorts. This shows the need to prioritize funding and support for prospective, multicenter trials, particularly those incorporating advanced endpoints such as neurocognitive outcomes, quality of life, LET-based biological modeling, and cost-effectiveness. Only through rigorous evaluation can the therapeutic value of proton therapy be definitively established and integrated into evidence-based treatment guidelines.

## 7. Limitations and strengths of study

The variability in assessment methods for adverse effects, neurocognitive function, and quality of life across studies poses a challenge. Standardization of assessment tools and criteria would enhance the comparability of results, providing a more robust basis for analysis. Moreover, the review’s dependence on English literature may introduce publication bias. Despite the limitations, this review reviews the existing literature on proton therapy for brain tumors.

## 8. Conclusion

This review sheds light on the evolving landscape of glioma treatment, focusing on the promising role of PBT. Gliomas, especially high-grade variants, present formidable challenges due to their aggressive nature and often limited treatment options. Traditional radiotherapy, while effective, poses concerns related to the nonselective nature of photon beams, leading to potential neurocognitive and neuroendocrine adverse effects. Proton beam therapy emerges as a promising alternative, leveraging the unique physical characteristics of protons to deliver precise radiation doses with reduced impact on surrounding healthy tissues. The positive outcomes highlighted in various studies, such as decreased neurocognitive decline, improved quality of life, and comparable tumor control rates, underscore the potential of PBT in revolutionizing glioma treatment. However, PBT outcomes include challenges and considerations. The observed neuroendocrine deficiencies and instances of radiation necrosis signal the need for continued research and management strategies to optimize PBT protocols. Balancing therapeutic efficacy and potential adverse effects is crucial, necessitating ongoing advancements in treatment planning algorithms, imaging technologies, and real-time monitoring tools. While this review consolidates valuable insights from diverse studies, it also identifies gaps and areas for future exploration. The limited focus on comprehensive clinical outcomes, the need for standardized assessment tools, and the challenges associated with cost and accessibility underscore the ongoing nature of research in this field.

## Author contributions

**Conceptualization:** Nicholas Aderinto.

**Writing – original draft:** Nicholas Aderinto, Gbolahan Olatunji, Emmanuel Kokori, Doyin Olatunji, Oluwatobi Omoworare, Adetola Emmanuel Babalola, Chimezirim Ezeano, Damilare Adewale Olusanya, David Timilehin Isarinade, Aminat Akinoso, Adeola Akinboade, Abdulrahmon Moradeyo, Naana Nkrumah-Ababio.

**Writing – review & editing:** Nicholas Aderinto, Gbolahan Olatunji, Emmanuel Kokori, Doyin Olatunji, Oluwatobi Omoworare, Adetola Emmanuel Babalola, Chimezirim Ezeano, Damilare Adewale Olusanya, David Timilehin Isarinade, Aminat Akinoso, Adeola Akinboade, Abdulrahmon Moradeyo, Naana Nkrumah-Ababio.
